# Efficacy and safety of ureterorenoscopy in the elderly: A systematic review axnd meta-analysis

**DOI:** 10.1371/journal.pone.0323237

**Published:** 2025-05-13

**Authors:** Minna Shen, Minqiang Shen

**Affiliations:** 1 Department of Surgery, Huzhou Third Municipal Hospital, the Affiliated Hospital of Huzhou University, Huzhou City, Zhejiang Province, China; 2 Department of Critical Care Medicine, Huzhou Third Municipal Hospital, the Affiliated Hospital of Huzhou University, Huzhou City, Zhejiang Province, China; Katholieke Universiteit Leuven UZ Leuven: Katholieke Universiteit Leuven Universitaire Ziekenhuizen Leuven, BELGIUM

## Abstract

**Objective:**

Ureterorenoscopy (URS) is a common procedure performed for renal or upper ureteric stones. Nevertheless, the efficacy and safety of URS in the elderly is unclear. We conducted the first meta-analysis of literature comparing outcomes of URS between elderly and non-elderly patients.

**Methods:**

Embase, PubMed, Web of Science, and Scopus databases were searched for studies relevant to the review. The last date was 2^nd^ September 2024. The elderly were defined as ≥ 65 or 60 years of age. Outcomes compared were stone-free rates (SFR), complications, and length of hospital stay (LOS).

**Results:**

Nine studies met the inclusion criteria. Pooled analysis showed that there was no difference in SFR between elderly and non-elderly groups after URS (OR: 0.96 95% CI: 0.81, 1.14 I^2^ = 3%). Meta-analysis failed to show any statistical significant in all complication rates (OR: 1.04 95% CI: 0.77, 1.40 I^2^ = 51%) as well as infectious (OR: 1.27 95% CI: 0.84, 1.92 I^2^ = 0%), medical (OR: 2.01 95% CI: 0.23, 17.57 I^2^ = 93%), surgical (OR: 1.18 95% CI: 0.68, 2.03 I^2^ = 0%) or Clavein Dindo grade ≥2 (OR: 1.02 95% CI: 0.60, 1.75 I^2^ = 0%) complications between elderly and non-elderly groups. Meta-analysis showed that the elderly had significantly longer LOS as compared to non-elderly patients (MD: 0.75 95% CI: 0.05, 1.45 I^2^ = 90%).

**Conclusions:**

URS seems to efficacious and safe in the elderly. Patients ≥60 or 65 years of age have similar SFR and complication rates as younger patients. However, LOS may be increased in the elderly. More robust studies taking into account baseline characteristics and importantly presenting rates are needed to validate the current results.

## Introduction

Urinary tract stone or urolithiasis is among the most common urological ailment seen in clinical practice [1]. The disease burden of urolithiasis is indeed high with some regional variation. Prevalence rates are about 0.1–14.8% in Western populations but can be as high as 10.6% in Asians [1,2]. Increasing the disability of the disease is the high recurrence rate with one in two patients suffering from repeated problems in the first decade of diagnosis [3]. Renal stones are also one of the common pain-related emergencies [4]. While most renal stone are asymptomatic or mild, a number of patients with large stones present with flank pain, infection, hydronephrosis, and diminished renal function [5]. The most common management protocols range from observation, expulsive therapy, extracorporeal shockwave lithotripsy (ESL), percutaneous nephrolithotomy (PCNL), and Ureterorenoscopy (URS). With the success of these modalities, open or laparoscopic surgical procedures are now only reserved for selected cases not treatable by minimally invasive therapies [6].

Over the years, URS has become the workhorse for the removal of renal and upper ureteric stones. Improvements in endoscopic technology have resulted in the production of thinner and flexible ureteroscopes with improved durability which has greatly expanded the indications of URS [7]. Research shows that URS may result in superior stone-free rates (SFR) when compared to ESL and fewer complications vs PCNL [8,9]. Published guidelines also indicate the URS can be safely used for removing stones up to 20mm in diameter with low complication rates [10,11]. Nevertheless, most studies assessing the safety and efficacy of URS have been conducted on the general population with predominantly middle-aged adults [12–14]. With a focus on preventive therapies and improved healthcare availability, the lifespan of humans has increased and most people are expected to live beyond 60 years of age. The World Health Organization statistics show that by 2030, one in every six individuals would be ≥ 60 years and by 2050, the world population of those ≥60 years will nearly double to 2.1 billion. Furthermore, a recent Japanese epidemiological study shows that the age of patients with upper urinary tract stones has shifted towards the elderly and it is expected that a higher number of older adults will undergo URS in the near future [15]. Therefore, the safety and efficacy of URS must be established in the elderly given the fact that such patients often have multiple comorbidities and are poor surgical candidates. In the past several studies have compared outcomes of URS for elderly vs non-elderly patients. However, no meta-analysis has been conducted to date. We therefore performed the present systematic review and meta-analysis to compare outcomes of URS between elderly and non-elderly patients.

## Materials and methods

This review is based on the reporting guidelines of PRISMA [16] (S1 File). The review protocol was registered on PROSPERO and can be accessed with the registration number CRD42024584819.

### Inclusion criteria

We considered cohort or case-control studies conducted on patients with renal stones undergoing URS for this review. Studies had to compare the outcomes of elderly vs non-elderly patients. For this review, the elderly was defined as either ≥60 or ≥ 65 years of age [17,18]. Outcomes needed for the review were SFR, complications, and length of hospital stay (LOS). We did not pre-define SFR and accepted all definitions of the included studies. Therefore, based on this, the PECOS eligibility criteria was:

Population: adult URS patients
Exposure: Elderly
Comparison: Non-elderly
Outcomes: SFR, complications, LOS
Study design: Cohort & Case-control

We excluded studies not including a non-elderly group, defining elderly with any other cut-off of age, and not reporting the required outcomes. Studies published as thesis and non-English language articles were also not included.

### Search strategy

Two reviewers screened repositories of Embase, PubMed, Web of Science, and Scopus for potential articles. The search included all articles from the inception of the respective databases to 2^nd^ September 2024. The search was restricted to human studies published as full-texts only. The review authors formulated the search strategy with the help of an experienced medical librarian. Details of the search can be found in S2 File. The search was further expanded to gray literature by screening Google Scholar. Additionally, the references of included papers were also screened.

### Study selection

The same two reviewers also conducted the selection of studies. In the initial step, all the articles retrieved by the authors from the searched databases were collated in a reference manager software [EndNote software (version X9.3.3)]. The software excluded all duplicate articles. In the second phase, the reviewers independently screened every article by evaluating its title and abstract. Any article selected by either reviewer for further evaluation was downloaded for further review. In the final step, both reviewers conducted the final selection of studies by reading the full texts. All disagreements were settled via consensus.

### Risk *of* bias analysis

The Newcastle Ottawa Scale (NOS) was used to estimate the risk of bias [19] by the two reviewers. All disagreements were resolved by consensus. Articles were evaluated for participant selection, comparability of groups, and outcomes. NOS gives four stars, two stars, and three stars for these areas, respectively. Nine is the maximum achievable score for the highest study quality.

### Data extraction

We prepared a table to reproduce all necessary study-related information. Information was obtained by two authors independently (Minna Shen and Minqiang Shen). Any disagreement was settled via consensus. Extracted data included the first author’s name, year of publication, country, study design, study groups, sample size, age, male gender, stone burden, multiple stones, location, type of ureteroscope, operating time, definition of SFR, and outcomes. We did not make any assumptions about missing data. In case of any clarity on data, the corresponding author was contacted once via email. If data was missing, the study was not included in the meta-analysis. Secondly, if the study reported multiple subgroups for elderly or younger populations, they were combined into single groups of elderly and non-elderly for this meta-analysis.

### Statistical analysis

We conducted a random-effects meta-analysis for all outcomes. Complication rates were reported in different ways by the included studies. After evaluating the reported data, the reviewers decided to pool data on all complication rates, infectious complications, medical complications, and surgical complications and a Clavien-Dindo grade of ≥ 2. All meta-analysis was conducted using “Review Manager” (RevMan, version 5.3) to generate odds ratios (OR) with 95% confidence intervals (CI) for dichotomous data and mean difference (MD) with 95% CI for continuous data. We used the Cochran Q test and I^2^ statistic to measure heterogeneity. Cut-offs were defined as follows: 0–25% values represented minimal heterogeneity, 26–75% moderate heterogeneity, and greater than 75% substantial heterogeneity. Publication bias was judged by assessing the symmetry of funnel plots. Sensitivity analysis was conducted for SFR and all complications by omitting individual studies one at a time. We also performed subgroup analysis for SFR and all complications based on the definition of elderly.

## Results

### Search results and baseline characteristics

**Fig 1:**
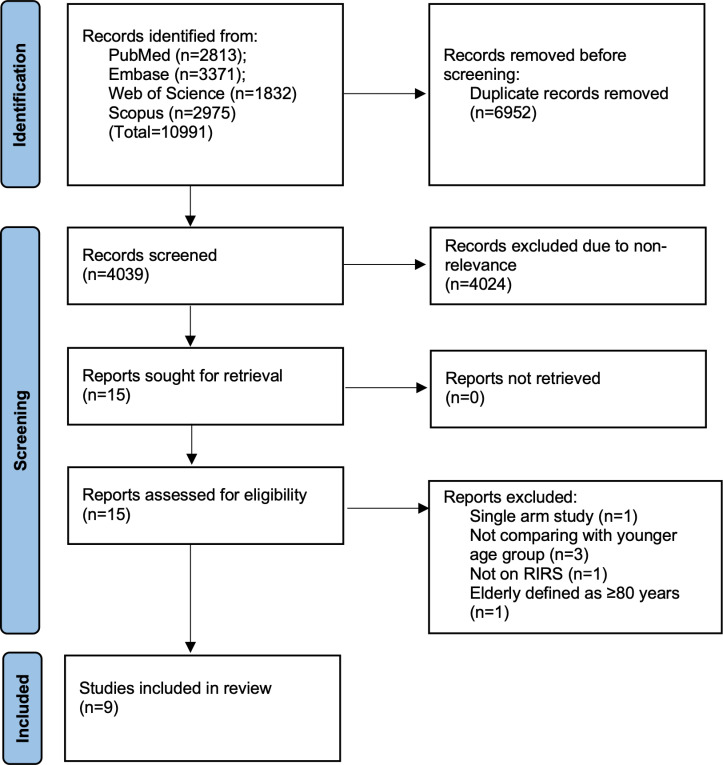
Study flowchart.

The flow of the search results is shown in the PRISMA flowchart (Fig 1). We retrieved 10991 articles initially. Most were duplicates and hence removed. 4039 articles underwent screening. Of them, 15 were selected for full-text review. Finally, nine studies met the inclusion criteria [20–28]. List of excluded studies with reasons is presented in S1 Table. No additional study was found in gray literature or reference lists. The inter-reviewer agreement for the selection of studies was perfect, without any discrepancies.

Study-specific details are shown in Table 1. The included studies were all retrospective cohort studies. Their country of origin was mostly Turkey and Japan. Several studies reported multiple subgroups of patients ≥60 or 65 years of age. These subgroups were combined into a single elderly group of ≥ 60 or 65 years to facilitate a meta-analysis. Correspondingly, non-elderly were defined as either <60 or < 65 years depending on the definition of the elderly (Table 1). Five studies defined the elderly as ≥ 65 years while the remaining used a definition of ≥ 60 years. The percentage of pre-operative stenting and the mean stone burden were variable in the included studies. However, none of the studies reported significant differences in the stone burden amongst the elderly and non-elderly groups. All studies used a rigid or flexible ureteroscope with a holmium YAG laser for accessing and fragmenting the stones. Studies used either plain radiographs, ultrasonography, or computed tomography for assessing SFR at 1–3 months of follow-up. The definition of SFR ranged from complete removal to residual stone of ≤ 2 or 4mm on follow-up. Most studies were of poor quality on NOS and received only a score of 6. This was because there was no adjustment of baseline factors between elderly and non-elderly groups in all except for one study [20] which received a score of 9.

**Table 1:** Details of included studies.

Study	Location	Groups (years)	n	Mean/ Median Age (years)	Males	Prop stenting (%)	Mean stone burden (mm)	Multiple stones (%)	Location- ureter (%)	Ureteroscope	Operating time (minutes)	Definition of stone-free status	NOS score^#^
Akgul 2024	Turkey	≥65	87	NR	55	40.2	822 ± 849^	35.7	23	Flexible with Holmium YAG laser	71.4 ± 30.9	RSF ≤ 23mm on CT	S**** C** O***
											66 ± 28.5		
			50-64	217		121	39.2	758 ± 737	30.9	26.3			
										60 ± 26.5			
										58.6 ± 26.4			
		30-49	324		201	32.1	739 ± 1033	38.9	22.2				
			18-29	69		42	27.5	456 ± 566	46.4	23.2			
Tamiya 2023	Japan	≥85	54	89	14	57.4	13.6 ± 10	25	NR	6.5/8.5-Fr or 6/7.5-Fr semi-rigid with Holmium YAG laser	69.5 ± 33.5	Complete removal or RSF ≤ 4mm on X-ray, USG, CT	S**** C O**
		75-84	172	78	86	40.7	14 ± 9.5	34.5			67.1 ± 34.5		
		65-74	316	69	195	26.6	16.8 ± 12.7	31.2			74.8 ± 37.1		
													75.3 ± 37.7
			<65	792	53	521	23.1	15.9 ± 12.2	30.9				
Taguchi 2022	Japan	≥85	27	87.8	4	81.5	11.4 ± 6.3	NR	62.9	6.5/8.5-Fr semi-rigid with Holmium YAG laser	84 ± 44.5	Complete removal or RSF < 2mm on CT	S**** C O**
											86.7 ± 44.7		
			65-85	81	71.2	35	58	10.8 ± 7.7		49.4			
										76.6 ± 35.1			
		<65	49	49	32	26.5	8.9 ± 4.3		65.3				
									56.7				
Koras 2021	Turkey	≥75	38	84	23	13.2	15 [10-20.5]	36.8	7.9	Rigid/flexible with Holmium YAG laser	50 [45-70]	Complete removal on X-ray or RSF ≤ 2mm on CT	S**** C O**
											49.5 [38.8-65] 45		
			60-75	230	62	113	22.6	14 [10–20]	31.7	17			
										[35-60]			
		<60	688	42	476	16.1	13 [10–20]	31.7	19.8				
Ozgur 2020	Turkey	≥60	33	65	18	NR	13 [9]	18.2	0	Flexible with Holmium YAG laser	40 [15]	RSF ≤ 2mm on CT	S**** C O**
		<60	45	43.3	30		14 [8]	17.8	0		40 [29]		
Cakici 2019	Turkey	≥60	291	67.2	159	NR	16.2 ± 7.5	18.6	17.2	NR	49 ± 18.1	Complete removal or RSF < 2mm on CT	S**** C O**
											45.3 ± 16.9		
			<60	1459	42	976		14.5 ± 7	17	22.4			
Berardinelli 2018	Europe	≥65	91	72.1	54	9.3	13 ± 5.8	34	NR	NR	62.3 ± 31.9	Complete removal or RSF < 2mm on CT	S**** C O**
		<65	308	48.6	202	15.8	12 ± 5.1	42.2			61.4 ± 30.5		
Yoshioka 2016	Japan	≥75	39	79.5	19	38.5	8.8 ± 3.2	18	7.7	12 Fr flexible with Holmium YAG laser	70.7 ± 30.6	Complete removal on X-ray	S**** C O**
		65-74	42	69.3	24	26.2	9.6 ± 3.3	33.3	7.1		72.4 ± 30.5		
		<65	108	51	73	29.6	9.5 ± 4.5	24	8.3		73.6 ± 29.96		
Tolga-Gulpinar 2015	Turkey	≥60	170	66.5	30	NR	17.2 ± 7.2	20	0	Flexible with Holmium YAG laser	53 ± 23.4	Complete removal or RSF ≤ 4mm on X-ray, USG, CT	S**** C O**
											49.3 ± 18.5		
			<60	726	41.4	424		15.8 ± 6.9	17.6	0			

Data was extracted by both reviewers (MS & MS) on 10 September 2024. Both reviewers confirm that every study was eligible for inclusion in the review.

RSF, residual stone fragments; USG, ultrasonography; CT, compute tomography; CaO, calcium oxalate; n, number of patients; Fr, French; NOS, Newcastle Ottawa scale; YAG, yttrium aluminum garnet; NR, not reported

^stone volume data

# Outcomes of NOS score are presented for the following domains: S, selection of cohort; C, comparability; O, outcome assessment. Stars in front of each abbreviation indicate the score of the study for the particular domain.

### Meta-analysis

**Fig 2:**
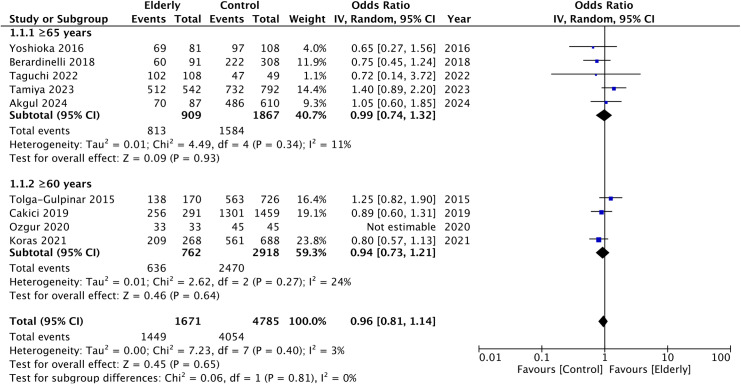
Meta-analysis of stone-free rates between elderly and non-elderly groups with subgroup analysis based on definition of elderly.

Data on SFR was reported by all included studies. Pooled analysis showed that there was no difference in SFR between elderly and non-elderly groups after URS (OR: 0.96 95% CI: 0.81, 1.14 I^2^ = 3%) (Fig 2). Subgroup analysis based on the definition of elderly also showed similar results for the subgroup of ≥ 65 years (OR: 0.99 95% CI: 0.74, 1.32 I^2^ = 11%) and ≥ 60 years (OR: 0.94 95% CI: 0.73, 1.21 I^2^ = 24%). There was no gross asymmetry on the funnel plot indicating the absence of publication bias (S1 Fig). Results also failed to change in significance on sensitivity analysis.

**Fig 3:**
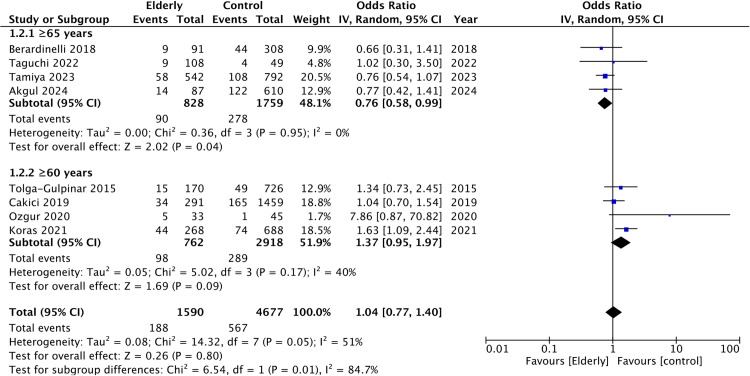
Meta-analysis of all complication rates between elderly and non-elderly groups with subgroup analysis based on definition of elderly.

Eight studies reported data on all complication rates. Meta-analysis failed to show any statistically significant difference between elderly and non-elderly groups (OR: 1.04 95% CI: 0.77, 1.40 I^2^ = 51%) (Fig 3). On subgroup analysis, the risk of complications was reduced in the elderly when defined as ≥ 65 years (OR: 0.76 95% CI: 0.58, 0.99 I^2^ = 0%) but not when defined as ≥ 60 years (OR: 1.37 95% CI: 0.95, 1.97 I^2^ = 40%). No change in the statistical significance of pooled effect size was noted on the sequential exclusion of studies.

**Fig 4:**
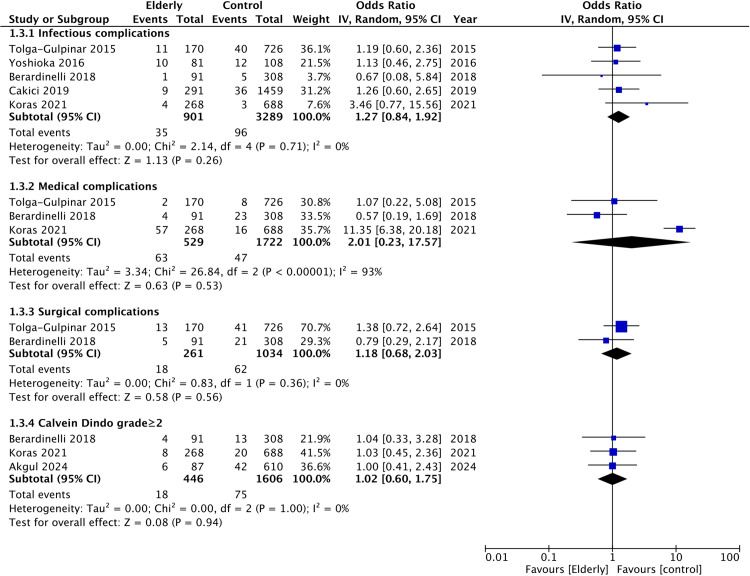
Meta-analysis of infectious, medical, surgical and Calvein Dindo grade ≥2 complication rates between elderly and non-elderly groups.

Examining different subtypes of complications, the meta-analysis did not show any statistically significant difference in infectious (OR: 1.27 95% CI: 0.84, 1.92 I^2^ = 0%), medical (OR: 2.01 95% CI: 0.23, 17.57 I^2^ = 93%), or surgical complications (OR: 1.18 95% CI: 0.68, 2.03 I^2^ = 0%). Three studies also reported Clavein Dindo grade ≥2 complication rates (defined as any complication requiring pharmacological or surgical intervention under local or general anesthesia). The meta-analysis showed no difference in the rates of Clavein Dindo grade ≥2 (OR: 1.02 95% CI: 0.60, 1.75 I^2^ = 0%) complications between the two groups (Fig 4).

**Fig 5:**
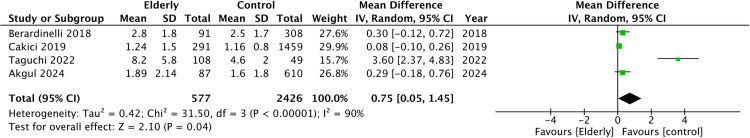
Meta-analysis of length of hospital stay between elderly and non-elderly groups.

LOS was reported only by four studies. Meta-analysis showed that the elderly had significantly longer LOS as compared to non-elderly patients (MD: 0.75 95% CI: 0.05, 1.45 I^2^ = 90%) (Fig 5).

## Discussion

Physiological alterations associated with aging and comorbidities often reduce the tolerance to surgical procedures [29]. Surgical interventions that may be deemed safe and effective in young and middle-aged adults may be morbid and even lethal in the elderly [30]. Therefore, the efficacy and safety of every surgical procedure must be established in the geriatric population to facilitate better outcomes [31]. The prevalence of urinary tract stones in the elderly seems to be rising [15]. Furthermore, the clinical presentation of urinary tract stones also differs between elderly and younger patients with the former demonstrating larger calculi, higher complication rates, and prolonged LOS after treatment [32]. Several studies have demonstrated that PCNL is efficacious and safe in the elderly with similar stone-free and complication rates as the younger age group [33–35]. However, similar conclusions cannot be drawn for URS which is a more invasive modality as compared to PCNL. Given this clinical dilemma, we conducted the first systematic review and meta-analysis of the literature to examine the efficacy and safety of URS in the elderly population. After a detailed literature search of four commonly accessed databases, we could identify nine studies comparing outcomes of URS between elderly and non-elderly populations. It must be clarified at the outset that there is no globally accepted definition of elderly. However, the two most commonly used cut-offs of age to identify the elderly used by the United Nations and World Health Organization are 60 and 65 years respectively [17,18]. Therefore, to facilitate a comprehensive review, both definitions were included in the present meta-analysis.

The primary measure of efficacy of any procedure for urinary tract stones is SFR. Indeed, elderly patients demonstrate several physiological changes that can affect the expulsion of the stone. Firstly, nephrosclerosis is inadequately compensated in geriatric patients leading to reduced urinary volume and glomerular filtration rate. Secondly, a reduction in the number of functional nephrons further reduces the glomerular filtration rate. Thirdly, peristalsis of the upper urinary system is reduced which may hinder stone expulsion after URS [36,37]. Nevertheless, the current meta-analysis showed that there was no difference in SFR between elderly and non-elderly patients undergoing URS. This indicates that the efficacy of URS is similar in both elderly as well as younger patients. Strengthening the outcome was the similarity of results on subgroup analysis based on the definition of the elderly. Secondly, the effect size remained stable on sensitivity analysis and no single study was noted to be an outlier.

Aging leads to several changes in the innate and adaptive components of the immune system leading to an increased risk of infections in the elderly. Further, the presence of comorbidities and multiple medications makes the perioperative management of the elderly challenging with an increased risk of perioperative and postoperative complications [30,38]. Studies have shown that the elderly undergoing stone treatment often have poor performance status, a higher prevalence of cardiovascular disorders, and several risk factors for postoperative sepsis [39]. The elderly also have more infection-related characteristics like a history of pyelonephritis, positive urine cultures preoperatively, and infectious stone compositions [21]. Nevertheless, our meta-analysis showed that the risk of complications after URS was not significantly different between elderly and non-elderly groups. This outcome was also stable on sensitivity analysis thereby increasing the credibility of the results. We also noted some contrasting results in the subgroup of studies using the cut-off of ≥ 65 years for defining the elderly, wherein a reduced risk of complications was noted in the elderly group. These results are to be interpreted with caution as only four studies were included in this subgroup. Secondly, we believe that the contrasting results could be related to the selection bias in these studies wherein the elderly received more rigorous and preventive care in anticipation of higher complication rates. To further explore different types of complications, the current review also analyzed infectious, medical, surgical, and Clavein Dindo grade ≥2 complication rates between the elderly and non-elderly groups. Herein too, no statistically significant increased risk of complications was noted in the elderly group thereby further validating the safety of URS in the elderly. However, the meta-analysis on LOS did show a significantly longer LOS in the elderly group. The results of this meta-analysis were primarily influenced by the study of Taguchi et al [22] as the remaining three studies did not find a significant difference in LOS between the elderly and non-elderly groups. Given the high heterogeneity in this meta-analysis, the results should be interpreted with caution.

While our review suggests that the age of ≥ 60 or 65 years does not affect the efficacy and safety of URS, it is still unclear how safe is the procedure in patients in the extremities of age. Two studies [21,22] in our review also examined patients ≥85 years of age and both noted no significant difference in SFR and complication rates between the oldest-old and younger patients. Likewise, Emilani et al [40] comparing URS patients ≥80 years and < 80 years have also noted no significant difference in SFR (71.4% vs 67.5%) and complication rates (9.5% vs 7.7%) between the two groups. To date, only one study by Drerup et al [41] has assessed the safety of URS in patients ≥90 years of age. They compared outcomes of 116 patients ≥90 years of age with 643 patients between 80–89 years of age and noted no difference in SFR (80% vs 79.8%) and intra-operative complication rates (6.6% vs 9.8%) between the two groups. The results of our review combined with the outcomes of these studies present important evidence supporting the use of URS in the geriatric age group.

There are certain limitations to this meta-analysis. Most importantly, only one study [20] minimized baseline differences between groups by propensity-score matching. All other studies had a low score on NOS indicating poor quality. Secondly, the number of studies available for review was not high. All were retrospective studies with a high chance of selection bias. Thirdly, data on types and grades of complications were not evenly reported by the studies. Clavein Dindo’s grades were reported just by three studies limiting a detailed analysis of the degree of complications. Fourthly, pre-operative stenting was more frequently used in elderly as compared to non-elderly patients in most studies. The safety of the procedure can be greatly influenced if patients are prestented prior to surgery. Because the prestenting rate is higher in older patients, this analysis confounds the issue of safety by age with the increased safety of presenting. Lastly, most data was from just two countries namely Turkey and Japan. While we cannot decipher any cause for regional bias of data, more studies from Japan could be due to the higher proportion of elderly in the latter. Due to limited data from Western populations and other Asian countries, the results should not be generalized till further data is available. A larger and more robust multi-centric study would provide deeper insights into the key objectives and offer more robust external validation.

## Conclusions

URS seems to be efficacious and safe in the elderly. Patients ≥60 or 65 years of age have similar SFR and complication rates as younger patients. However, LOS may be increased in the elderly. More robust studies taking into account baseline characteristics and importantly presenting rates are needed to validate the current results.

## Supporting information

S1 Fig Funnel plot of the meta-analysis of stone-free rates. (PDF)

S1 Table List of excludes studies. (DOCX)

S1 File PRISMA flowchart. (DOCX)

S2 File Search strategy. (DOCX)

S3 File Articles included in the meta-analysis. (RAR)
