# Supplementary figures and images for: Efficacy and safety of ureterorenoscopy in the elderly: A systematic review axnd meta-analysis
Source: PLoS One. 2025 May 13;20(5):e0323237. doi: 10.1371/journal.pone.0323237 (PMC12074608; doi:10.1371/journal.pone.0323237)

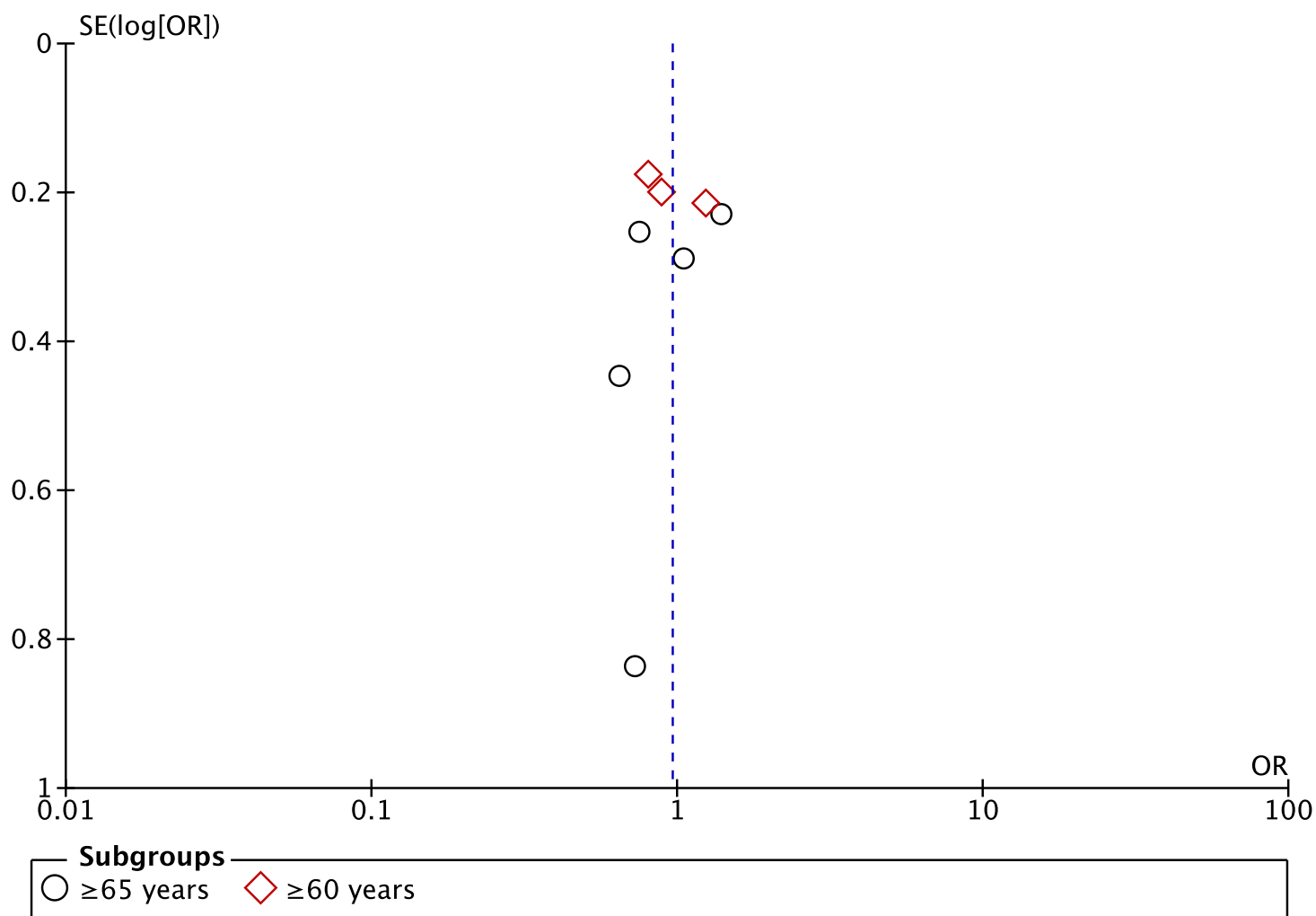

Supplement: S1 Fig — (PDF) [file pone.0323237.s001.pdf]
