# Supplementary material for: Efficacy and safety of ureterorenoscopy in the elderly: A systematic review axnd meta-analysis
Source: PLoS One. 2025 May 13;20(5):e0323237. doi: 10.1371/journal.pone.0323237 (PMC12074608; doi:10.1371/journal.pone.0323237)
Supplement: S1 Table — (DOCX) [file pone.0323237.s002.docx]

S1 Table: List of excluded studies

| Excluded study | Reason |
| --- | --- |
| Prattley et al[1] | Single arm study |
| Aykac et al[2] | Not comparing with younger age group |
| Drerup et al[3] | Not comparing with younger age group |
| Giulioni et al[4] | Not comparing with younger age group |
| Mager et al[5] | Not on URS |
| Emilani et al[6] | Elderly defined as ≥80 years |

References

1. Prattley S, Voss J, Cheung S, Geraghty R, Jones P, Somani BK. Ureteroscopy and stone treatment in the elderly (≥70 years): prospective outcomes over 5- years with a review of literature. Int Braz J Urol. 2018;44: 750–757. doi:10.1590/S1677-5538.IBJU.2017.0516

2. Aykac A, Baran O. Safety and efficacy of retrograde intrarenal surgery in geriatric patients by age groups. Int Urol Nephrol. 2020;52: 2229–2236. doi:10.1007/s11255-020-02564-1

3. Drerup M, Özsoy M, Wehrberger C, Lenz M, Ramesmayer C, Stolzlechner P, et al. Ureteral calculi in octogenarians and nonagenarians: Contemporary in-hospital management-A joint study by the endourological section of the Austrian Association of Urology. PLoS One. 2023;18: e0280140. doi:10.1371/journal.pone.0280140

4. Giulioni C, Brocca C, Gauhar V, Somani BK, Chew BH, Traxer O, et al. Does age impact outcomes of retrograde intrarenal surgery in the elderly? Results from 366 patients from the FLEXible ureteroscopy outcomes registry (FLEXOR). Aging Clin Exp Res. 2023;35: 2711–2719. doi:10.1007/s40520-023-02545-1

5. Mager R, Brauers C, Kurosch M, Dotzauer R, Borgmann H, Haferkamp A. Outcomes for Geriatric Urolithiasis Patients aged ≥80 Years Compared to Patients in Their Seventies. Eur Urol Focus. 2022;8: 1103–1109. doi:10.1016/j.euf.2021.08.004

6. Emiliani E, Piccirilli A, Cepeda-Delgado M, Kanashiro AK, Mantilla D, Amaya CA, et al. Flexible ureteroscopy in extreme elderly patients (80 years of age and older) is feasible and safe. World J Urol. 2021;39: 2703–2708. doi:10.1007/s00345-020-03448-w
