# Supplementary material for: Efficacy and safety of ureterorenoscopy in the elderly: A systematic review axnd meta-analysis
Source: PLoS One. 2025 May 13;20(5):e0323237. doi: 10.1371/journal.pone.0323237 (PMC12074608; doi:10.1371/journal.pone.0323237)
Supplement: S2 File — (DOCX) [file pone.0323237.s004.docx]

S2 File: Search strategy

**PubMed :**

(((((elderly) OR (aged)) OR (old)) OR (older)) OR (senile)) AND (((Retrograde Intrarenal Surgery[Title/Abstract]) OR (Ureteroscopy[Title/Abstract])) OR (ureterorenoscopy[Title/Abstract]))

**Embase :**

1. ‘Elderly’

2. ‘Old’

3. ‘Aged’

4. ‘senile’

5. ‘retrograde intrarenal surgery’

6. ‘ureteroscopy’

7. ‘ureterorenoscopy’

8. #1 OR #2 OR #3 OR #4

9. #5 OR #6 OR #7

10. #8 AND #9

**Web of Science AND Scopus:**

(((((elderly) OR (aged)) OR (old)) OR (older)) OR (senile)) AND (((Retrograde Intrarenal Surgery) OR (Ureteroscopy)) OR (ureterorenoscopy))
